# Analyzing the Possibility of Utilizing CBCT Radiomics as an Independent Modality: A Phantom Study

**DOI:** 10.31557/APJCP.2021.22.5.1383

**Published:** 2021-05

**Authors:** Dharmendran Palani, Senthilkumar Shanmugam, Kesavan Govindaraj

**Affiliations:** 1 *Research and Development Centre, Bharathiar University, Coimbatore, India. *; 2 *Department of Radiotherapy Government Rajaji Hospital & Madurai Medical College, Madurai, Tamil Nadu, India. *; 3 *Department of Radiotherapy, Vadamalayan Hospitals Integrated Cancer Centre, Madurai, India. *

**Keywords:** Texture, quantitative imaging features, cone-beam CT, computed tomography

## Abstract

**Aim:**

To verify if computed tomography (CT) radiomics were reproducible by cone beam CT (CBCT) radiomics by using Catphan^®^ 504.

**Materials and Methods:**

Catphan^®^ 504 was imaged using the default IGRT OBI CBCT imaging protocols and CT scanner. Seven known density image regions of the phantom were segmented and image feature was extracted by Imaging Biomarker Explorer (IBEX) software. The 49 selected features from four feature categories were analyzed by considering each region of interest (ROI) segment as individual image set. Correlation was studies using interclass correlation coefficient (ICC) and Pearson’s correlation coefficient.

**Results:**

The ICC of the three feature categories, namely intensity, GLCM, and GLRLM was significant (p-value<0.05) in comparison with CT, while the ICC of the fourth feature category, NID, was no significant. The average absolute Pearson’s correlation coefficient from the features of the images was as follows: CT: r=0.679±0.257, CBC_Thead_: r=0.707±0.231, CBCT_thorax_: r=0.643±0.260, and CBCT_pelvis_: r=0.594±0.276.

**Conclusion:**

It seems that the various densities of Catphan^® ^504 ROI image segments of the CT radiomics are reproducible with CBCT radiomics and CBCT radiomics can be used as an independent modality.

## Introduction

The field of medical imaging provides comprehensive imaging tools. The applications of multimodality images in radiotherapy development from the recent past to the current diagnostic images were appreciated in precise delineation of target volume (TV), organ at risk (OAR), treatment plan, treatment response assessment and treatment follow up. Amongst the various multimodality images, computed tomography (CT) images plays two vital roles, namely defining pretreatment tumor and tumor responses to treatment, of which the later impacts greatly the treatment decision. However, the recent modern advancements in imaging analysis, namely radiomics, extracts additional quantitative features from medical images, such as CT, positron emission tomography (PET), and magnetic resonance imaging (MRI), to uncover patient’s response to treatment as well as the chance of developing side effects (Gillies et al., 2016). CT radiomics is a conventionally practiced prognostic feature in radiation therapy. Various clinical, phantom, and texture analysis of CT radiomics have discussed both its reliability in assessing tumor response to treatment and its limitations. However, such limitations outweigh the established purpose of CT radiomics (Nardone et al., 2016; Nie et al., 2016; De Cecco et al., 2016; Bundschuh et al., 2014; Pyka et al., 2015; Tian et al., 2015; Yip et al., 2014). Three - dimensional (3D) cone –beam CT (CBCT) is taken regularly for patient setup and position verification (Lambin et al., 2017). These images are then used to study the tumor changes during the course of treatment (Brink et al., 2014). Radiomics based on CBCT imaging offers a potential for primary treatment process where the prognostic value of conventional CT images is already known (Fried et al., 2014; Coroller et al., 2015; Aerts et al., 2014). There are very few literatures explaining the possibilities for image features extracted from CBCT to be a potential alternative for CT. Moreover, the prospective of radiomics in CBCT needs to be investigated particularly in the image quality of CBCT as this dimension is unfamiliar when compared to the conventional CT. Therefore, this study aimed to assess the quality of CBCT-based radiomic features with selected known image density region of interest (ROIs) and compared to it CT-based radiomics features. The parameter of interest was to analyze the gray level value changes of the ROIs image density between the CBCT and CT by computing the first order intensity and texture matrices. To assess the reliability of CBCT radiomic features, Catphan^®^ 504 phantom images of head, thorax, and pelvis image guided radiation therapy (IGRT) protocols of on-board imaging (OBI) CBCT were obtained since phantoms have proved to provide robustness in non-invasive studies .This study further investigated the possibility of utilizing CBCT radiomics as an independent modality to assess the tissue equivalent density region.

## Materials and Methods

*Catphan* ^®^ *504*

Catphan^®^ 504 phantom is the most common available phantom in IGRT. It is supplied by Varian Medical Systems. This phantom is designed to monitor sensitometry target values. Over the time, it is proved to provide valuable information like indicating changes in scanner performance. It is regularly used to define the CT density table in the treatment planning system for patient dose calculation and as OBI CBCT image calibration . Catphan® 504 phantom module has sensitomerty region which has known density material inserts made up of Teflon, Delrin, acrylic, polystyrene, low density polyethylene (LDPE), polymethylpentene (PMP), and air. The relative electron densities are 1.868, 1.363, 1.147, 0.998, 0.945, 0.853, and 0.001, respectively. Each density insert is 1.25 cm diameter and 2.5 cm in length. This phantom was chosen for this study for its predefined known seven ROI density and easy accessibility in most of the IGRT setup as it is routinely used as a tool for determining image calibration and quality. 

*Phantom imaging*

To determine the similarity between the CBCT radiomics texture values and those of CT images, we imaged Catphan® 504 using the default IGRT head ,thoracic and pelvis CBCT Varian Linac OBI imaging protocols and GE medical system (Discovery IQ) CT scanner. Each CBCT scan is classified as CBCT head scan (CBCThead), CBCT thorax scan (CBCT thorax), and CBCT pelvis scan (CBCT pelvis). The characteristics of the scan parameters are given in Table 1.

*ROI segmentation*

**Figure 1:**
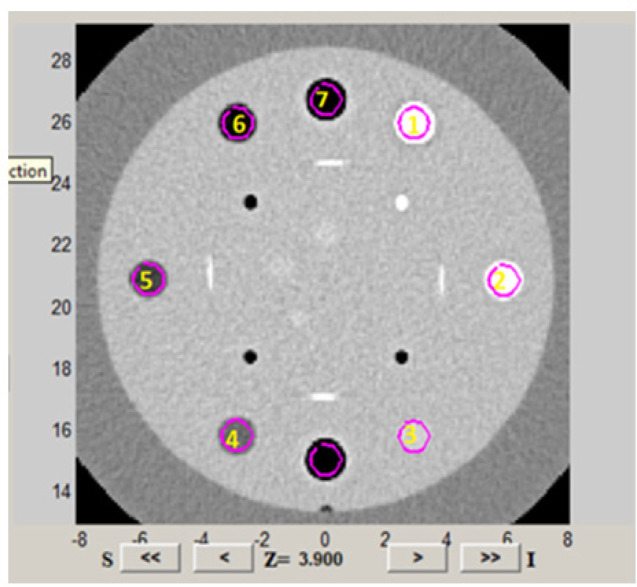
Axial Section Taken for the Analysis of CT Catphan® 504 Sensitometry Region Image with Segmented ROI Known Density Materials 1)Teflon , 2)Delrin , 3) Acrylic ,4) Polystyrene , 5) LDPE, 6) PMP and 7) Air

**Figure 2:**
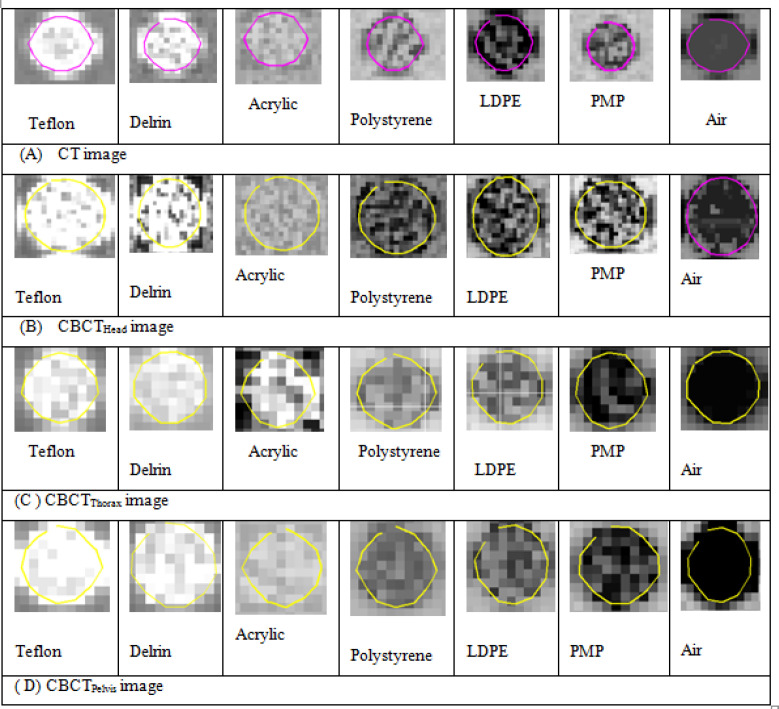
Different ROI Density Sensitomerty Regions Catphan® 504 (A) CT Image (B)-(D) CBCT Images

The scanned images in Digital Imaging and Communication in Medicine (DICOM) format were imported into Imaging Biomarker Explorer (IBEX) software (available for download at http://bit.ly/ IBEX_MDAnderson). ROI density was delineated in all the four scanned image sets using the IBEX graphical user interfaces (GUI) countering option. Delineated segmented ROI shown in Figure1 were considered to be sub images for the sources of texture feature extraction. Each ROI was manually segmented and added to the data sets which contained basic image information, such as ROI statistics, voxel, ROI contours, ROI binary masks, and image data in the ROI bounding box. As there were possibilities of inaccurate contour values, accurate countering was necessary and features were only calculated within these areas. Radiomics features extracted from the segmented density ROI volume within phantom is highlighted in Figure 2.

*Radiomics feature definition and extraction*

ROI data of four image sets were preprocessed with histogram equalization enhancement preprocessing algorithm. The four-feature categories and 49 features extraction algorithm were developed in the feature algorithm workspace in IBEX. The selected 49 features can be generated for different applications, such as tumor diagnosis, tumor staging, gene prediction, and outcome prediction. 

The selected feature categories for this phantom study were 11 Intensity Histogram and Gray Level Run Length Matrix, 22 Gray level Co-occurrence Matrix, and 5 Neighborhood Intensity Difference matrix, as shown in the Table 2. 

*Statistical analysis*

The feature category clusters, namely intensity, GLCM, GLRLM, and NID radiomics feature values, extracted from individual density ROIs image data set were selected for the analysis. The interrelation relatability between the images was calculated using intra-class correlation coefficient (ICC). Variance estimates were obtained using two-way mixed effects and absolute agreement method. The ICC is a statistical measure ranging from 0 to 1, indicating null and perfect reproducibility, respectively. The ICC > 0.7 is considered significant in the interrelated reproducibility between the CT and CBCTs. The bivarFiate Pearson’s correlation coefficient was used to analysis the density dependent related changes of the extracted radiomics features from the known seven ROIs present within the CT and CBCT. The correlation coefficient value ranges from ±1 to 0 in which -1 and +1 indicates perfect negative and perfect positive, respectively. In this condition, r=0 (zero relationship) implies no correlation, 0.1< |r| < ±0.3 is said to show small / weak correlation, while the values between ±0.3< |r| < ±0.5 medium / moderate correlation and ±0.5<|r| is large / strong correlation. In this study, the ICC and Pearson’s correlation coefficient were analyzed using the IBM SPSS.

## Results

The radiomics features extracted using IBEX from seven known phantom density ROIs image data set were concurrently analyzed with CBCT inter scan IGRT imaging protocol ROIs image segment radiomics feature category cluster reproducibility, and radiomics feature density dependency consistency was compared with known CT features.

*CBCT interrelation relatability with CT image*

**Figure 3:**
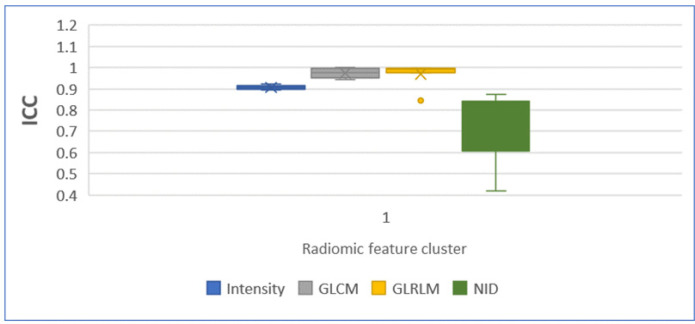
Box Plot Wispier of Radiomics Feature Cluster vs. ICC

Radiomics feature category clusters, namely intensity, GLCM, GLRLM, and NID, were extracted from the seven density ROIs segments. Each ROI was considered as individual image feature data set. The ICC was calculated for the ROI feature category clusters of CBCT and CT images, and the results are expressed in Table 3. The average ICC of the feature category cluster was significant (ICC=0.913±0.123, P=0.011). Correlation value more than > 0.7 is considered as strong interrelated repeatability among the images. ICC analysis result of the first three radiomics cluster features indicated significant CBCT inter scan IGRT imaging protocol interrelated repeatability with CT. However, NID cluster feature value from acrylic and air ROIs had ICC=0.418 (P=0.209) and ICC=0.607 (P=0.09), respectively, revealing less correlation significance. Each ROI segment cluster feature category ICC comparison is shown in Figure 3. 

*ROI density radiomics feature correlation consistency among the images*

 The 49 computed features correlation strength significance and magnitude were compared among the images. Here, the Pearson’s scoring of the four image sets, namely CT, CBCThead , CBCTthorax ,and CBCTpelvis, was considered to be the similarity predictors. The average absolute Pearson’s correlation coefficient from the features of the images was as follows: CT: r=0.679±0.257 (40 of the 49 features had r>0.5 and 55% of the selected features had significant (p-value<0.05)), CBCT_head_: r=0.707±0.231 (39 of the 49 features had r>0.5 along with 59% of the selected showed the significance (p-value <0.05)), CBCT_thorax_ : r=0.643±0.260 (35 of the 49 features had r>0.5 with 43% significance (p-value <0.05)), and CBCT_pelvis_ :r=0.594±0.276 (27 of the 49 features had r>0.5 and 41% significance (p-value <0.05)). Nineteen computed features out of the 49 selected radiomics features from all the four image sets had very strong correlation (r > 0.750) and were significant (p-value<0.05) as shown in the Figure 4(a-d). The results of Pearson’s correlation coefficient test on the image radiomics features are presented in Table 4 (a-d).

**Table 1:** Characteristics of the Scan Parameters of the CT and CBCT Images

Manufacturer	GE medical system CT	Varian medical system OBI		
Scan protocol	CT-Pelvis	CBCT-Head	CBCT Thorax	CBCT Pelvis
Image size (pixels)	512x512	384x384	384x384	384x384
Pixels size (mm)	0.0976	0.0625	0.1171	0.1171
Slice thickness (mm)	2.5	2.5	2.5	2.5
Tube Voltage (kVp)	120	100	110	125
Exposure time (mAs)	6	145	262	680
Tube current (mA)	300	20	20	80
Scanner rotation	Helical	Half fan 200º	Full fan 360º	Full fan360º
Filter		Full bowtie filter	Half bowtie filter	Half bowtie filter
No of image Projection		360	655	655

**Table 2:** Feature Extraction Algorithms Used in the Study with Estimated Feature Names

Feature type	Feature Category	Estimated Feature Names	Comment
First order Intensity	Intensity	Energy, Entropy, Max, Mean, Median, Min, standard deviation, Uniformity, Kurtosis, Skewness and Variance	
Texture	Gray level co-occurrence matrix25 (GLCM)	Autocorrelation , Cluster Prominence Cluster Shade , Cluster Tendency , Contrast Correlation , Difference Entropy , Dissimilarity , Energy , Entropy , Homogeneity, Homogeneity2, Information Measure Correlation1, Information Measure Correlation 2 , Inverse Difference Moment Normalised, Inverse Difference Normalised , Inverse Variance, Max Probability, Sum Average, Sum Entropy Sum Variance and Variance	25:= GLCM is computed from all 2D image slices
	Gray level run length matrix (GLRLM)	Gray Level Non-uniformity, High Gray Level Run Emphasis, Long Runs Emphasis , Long Run High Gray Level Emphasis, Long Run Low Gray Level Emphasis , Low Gray Level Run Emphasis, Run Length Non-uniformity, Run Percentage, Short Runs Emphasis, Short Run High Gray Level Emphasis, Short Run Low Gray Level Emphasis.	
	Neighborhood intensity difference matrix25 (NID)	Busyness, Coarseness, Complexity, Contrast and Texture Strength	25:= Neighborhood intensity different (NID) is computed from all 2D image slices

**Table 3:** ICC Values Comparsion between CT and CBCT Radiomics Images

Feature type	Phantom image ROI segment Density materials	Average measures Intra class Correlation CT with CBCT radiomic features	Significances p-value	95% Confidence Interval Lower Bound	Upper Bound
Intensity	Teflon	0.902	0	0.787	0.963
	Delrin	0.908	0	0.800	0.965
	acrylic	0.895	0	0.773	0.961
	Polystyrene,	0.896	0	0.774	0.961
	LDPE	0.922	0	0.83	0.970
	PMP	0.914	0	0.812	0.967
	Air	0.91	0	0.805	0.966
Texture GLCM	Teflon	0.952	0	0.909	0.978
	Delrin	0.977	0	0.956	0.989
	acrylic	0.999	0	0.997	0.999
	Polystyrene,	0.995	0	0.990	0.998
	LDPE	0.99	0	0.980	0.995
	PMP	0.943	0	0.892	0.974
	Air	0.958	0	0.921	0.981
Texture GLRLM	Teflon	0.994	0	0.986	0.998
	Delrin	0.995	0	0.989	0.999
	acrylic	0.976	0	0.941	0.993
	Polystyrene,	0.988	0	0.971	0.996
	LDPE	0.996	0	0.991	0.999
	PMP	0.997	0	0.992	0.999
	Air	0.845	0	0.619	0.953
Texture NID	Teflon	0.874	0.002	0.519	0.985
	Delrin	0.827	0.008	0.343	0.980
	Acrylic	0.418*	0.209	-1.230	0.933
	Polystyrene,	0.699	0.047	-0.144	0.965
	LDPE	0.824	0.008	0.332	0.980
	PMP	0.84	0.006	0.391	0.982
	Air	0.607*	0.093	-0.491	0.954

**Figure 4a:**
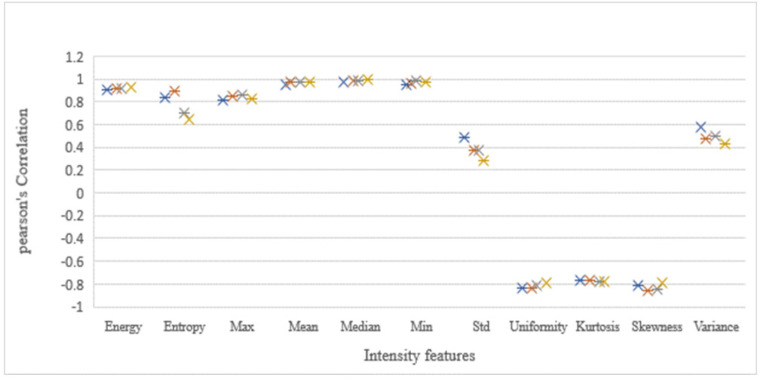
Intensity Feature Relationship was Compared with Density Pearson’s Correlation Coefficient in the Four Images and the Correlation Significances were Above r=0.75 and below r=-0.75

**Table 4a:** Radiomics Feature Variability with Respect to ROI Segment Density in Intensity Feature

Intensity features	CT		CBCT_head _		CBCT_thorax_		CBCT_pelvis_	
	Pearson (r)	Sig p-value	Pearson (r)	Sig p-value	Pearson (r)	Sig p-value	Pearson (r)	Sig p-value
^a^Energy*	0.900	0.006	0.916	0.004	0.913	0.004	0.925	0.003
Entropy*	0.837	0.019	0.897	0.006	0.699	0.081	0.647	0.116
^a^Max*	0.810	0.027	0.854	0.014	0.858	0.014	0.827	0.022
^a^Mean*	0.952	0.001	0.973	0.000	0.976	0.000	0.977	0.000
^a^Median*	0.969	0.000	0.98	0.000	0.986	0.000	0.990	0.000
^a^Min*	0.946	0.001	0.965	0.000	0.985	0.000	0.970	0.000
Std	0.488	0.267	0.370	0.413	0.370	0.414	0.283	0.539
^a^Uniformity*	-0.838	0.019	-0.841	0.018	-0.811	0.027	-0.795	0.033
^a^Kurtosis*	-0.764	0.046	-0.766	0.045	-0.774	0.041	-0.774	0.041
^a^Skewness*	-0.813	0.026	-0.854	0.015	-0.845	0.017	-0.794	0.033
Variance	0.582	0.170	0.475	0.282	0.498	0.255	0.428	0.338

^a^, indicates that the feature from the four image modalities had a strong correlation with significant p-value<0.05; *, indicates that the feature from the four image modalities had a strong correlation with r>0.5

**Table 4b:** Radiomics Texture Feature Variability with Respect to ROI Segment Density in GLCM Feature

Gray Level Co occurrence Matrix feature	CT		CBCT_head _		CBCT_thorax_		CBCT_pelvis_	
	Pearson (r)	Sig p-value	Pearson (r)	Sig p-value	Pearson (r)	Sig p-value	Pearson (r)	Sig p-value
^a^Auto Correlation*	0.903	0.005	0.925	0.003	0.92	0.003	0.926	0.003
Cluster Prominence*	0.610	0.146	0.601	0.154	0.609	0.146	0.553	0.198
^a^Cluster Shade*	-0.859	0.013	-0.844	0.017	-0.832	0.020	-0.903	0.005
Cluster Tendency	0.546	0.205	0.516	0.236	0.526	0.226	0.427	0.339
Contrast	0.642	0.120	0.410	0.36	0.438	0.326	0.45	0.311
Correlation	-0.231	0.618	0.565	0.186	0.482	0.273	0.088	0.851
Difference Entropy	0.729	0.063	0.639	0.122	0.378	0.403	0.299	0.514
Dissimilarity	0.701	0.08	0.417	0.352	0.441	0.321	0.389	0.389
^a^Energy*	-0.806	0.028	-0.815	0.026	-0.797	0.032	-0.768	0.044
Entropy*	0.784	0.037	0.883	0.008	0.638	0.123	0.571	0.180
Homogeneity	-0.782	0.038	-0.877	0.01	-0.604	0.151	-0.449	0.313
Homogeneity2	-0.758	0.049	-0.882	0.009	-0.582	0.171	-0.411	0.359
InformationMeasureCorr1	0.169	0.718	-0.548	0.203	-0.238	0.607	-0.272	0.555
InformationMeasureCorr2	0.153	0.744	0.581	0.171	0.299	0.515	0.256	0.580
Inverse Diff Moment Norm	-0.642	0.12	-0.403	0.37	-0.434	0.330	-0.445	0.317
Inverse Diff Norm	-0.706	0.076	-0.42	0.348	-0.441	0.322	-0.378	0.404
Inverse Variance	0.164	0.725	0.292	0.526	0.042	0.929	0.154	0.742
^a^Max Probability*	-0.803	0.03	-0.808	0.028	-0.802	0.03	-0.783	0.037
^a^Sum Average*	0.951	0.001	0.974	0.000	0.977	0.000	0.977	0.000
Sum Entropy*	0.806	0.029	0.814	0.026	0.631	0.129	0.557	0.194
^a^Sum Variance*	0.902	0.006	0.922	0.003	0.919	0.003	0.926	0.003
Variance	0.546	0.205	0.516	0.236	0.526	0.226	0.427	0.339

^a^, indicates that the feature from the four image modalities had a strong correlation with significant p-value<0.05; *, indicates that the feature from the four image modalities had a strong correlation with r>0.5

**Table 4c:** Radiomics Texture Feature Variability with Respect to ROI Segment Density in GLRLM Feature

Gray Level Run Length Matrix Features	CT		CBCT_head _		CBCT_thorax_		CBCT_pelvis_	
	Pearson (r)	Sig p-value	Pearson (r)	Sig p-value	Pearson (r)	Sig p-value	Pearson (r)	Sig p-value
Gray Level Non uniformity	-0.044	0.925	-0.76	0.048	0.072	0.879	-0.260	0.573
^a^High Gray Level Run Emphasis*	0.883	0.008	0.906	0.005	0.906	0.005	0.911	0.004
Long Run Emphasis*	-0.797	0.032	-0.809	0.028	-0.802	0.030	-0.737	0.059
Long Run High Gray Level Emphasis	-0.316	0.490	0.725	0.065	0.658	0.108	0.772	0.042
^a^Long Run Low Gray Level Emphasis*	-0.820	0.024	-0.827	0.022	-0.821	0.024	-0.832	0.020
^a^Low Gray Level Run Emphasis*	-0.989	0.000	-0.955	0.001	-0.944	0.001	-0.948	0.001
Run Length Non uniformity	0.721	0.068	0.862	0.013	0.697	0.082	0.483	0.272
Run Percentage*	0.767	0.044	0.836	0.019	0.765	0.045	0.608	0.147
Short Run Emphasis	0.749	0.053	0.857	0.014	0.621	0.136	0.418	0.351
^a^Short Run High Gray Level Emphasis*	0.894	0.007	0.906	0.005	0.893	0.007	0.897	0.006
^a^Short Run Low Gray Level Emphasis*	-0.816	0.025	-0.980	0.000	-0.966	0.000	-0.965	0.000

^a^, indicates that the feature from the four image modalities had a strong correlation with significant p-value<0.05; *, indicates that the feature from the four image modalities had a strong correlation with r>0.5

**Figure 4b:**
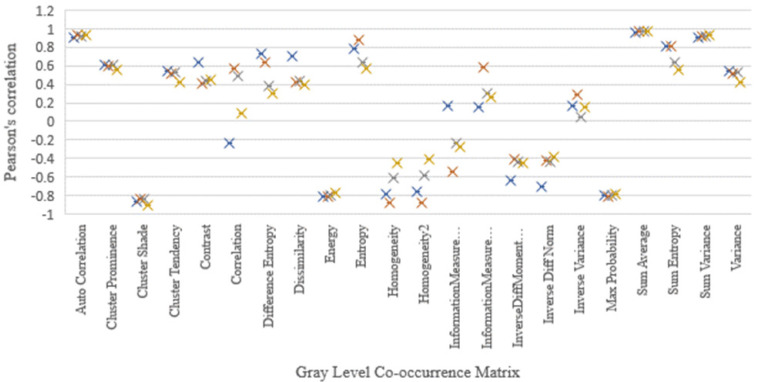
GLCM Feature Relationship was Compared with Density Pearson’s Correlation Coefficient in the Four Images and the Correlation Significances were Above r=0.75 and below r=-0.75

**Table 4d:** Radiomics Texture Feature Variability with Respect to ROI Segment Density in NID Feature

Neighbor Intensity Difference Feature	CT		CBCT_head _		CBCT_thorax_		CBCT_pelvis_	
	Pearson (r)	Sig p-value	Pearson (r)	Sig p-value	Pearson (r)	Sig p-value	Pearson (r)	Sig p-value
Busyness	0.07	0.881	-0.453	0.307	0.031	0.947	-0.237	0.609
Coarseness	-0.787	0.036	-0.244	0.599	-0.603	0.152	-0.129	0.783
Complexity*	0.68	0.093	0.549	0.202	0.658	0.108	0.54	0.211
Contrast	0.743	0.056	-0.048	0.918	0.669	0.1	0.468	0.289
Texture Strength	-0.103	0.827	0.428	0.338	0.121	0.796	0.06	0.899

^a^, indicates that the feature from the four image modalities had a strong correlation with significant p-value<0.05; *, indicates that the feature from the four image modalities had a strong coefficient with r>0.5

**Figure 4c:**
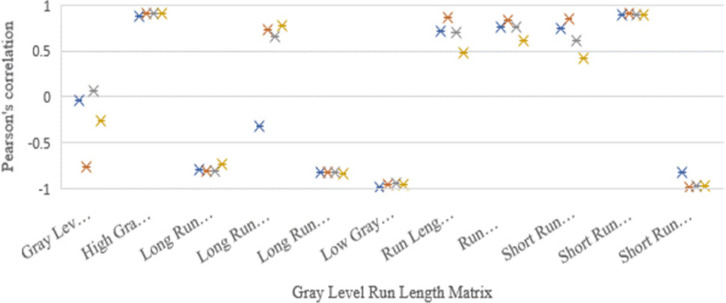
GLRLM Feature Relationship was Compared with Density Pearson’s Correlation Coefficient in the Four Images and the Correlation Significances were Above r=0.75 and Below r=-0.75

**Figure 4d:**
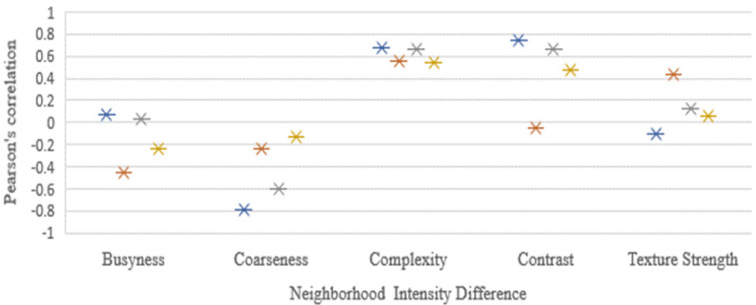
NID Feature Relationship was Compared with Density Pearson’s Correlation Coefficient in the Four Images and the Correlation Significances were Above r=0.75 and Below r=-0.75

## Discussion

In this phantom study, amongst the four inter scan image modalities, CBCThead radiomics features, 80% of the 49 selected features demonstrated strong correlation r>0.5, and 55% of the features were significant (p-value <0.05). Similarly, other two modalities of CBCTthorax and CBCTpelvis had 10% less and below of CBCThead. The IGRT CBCT image reconstruction filters differed from their imaging protocols. This significant difference in the CBCT images might be due to the image reconstruction (Zhao et al., 2014; Rizz et al., 2005).

The results on the extracted four radiomics feature categories from the image sets of the phantom were as follows. Out of the 11 intensity features, 8 showed significant correlation with CT. The skewness and kurtosis of the intensity features category are used as a prognostic tools in identifying tumor genetic mutation of NSCLC (Weiss et al., 2014), while another study used skewness to predict overall survival (Ahn et al., 2015). Image features extracted from CBCT also act as an early potential biomarker for treatment response assessments (Bertelsen et al., 2011; Bernchou et al., 2015). Similarly, 5 of the 11 selected texture features of GLRLM had significant correlation with CT. The GLRLM feature expresses its usefulness in distinguishing benign lymph nodes from malignant ones (Bayanati et al., 2015). On the other hand, among the 22 GLCM features, 9 had strong correlation with CT, which is in line with findings of a previous study calculated the CT images using an anthropomorphic which phantom (Mahmood et al., 2017).However, in a clinical study, Coroller et al., considered this feature to the best radiomics feature in predicting distant metastasis in NSCLC patients (Coroller et al., 2015). Finally, out of 5 NID features, only one feature showed strong correlation with CT, which is in accordance with findings of Mahmood et al.’s study. Thus, we found that the intensity and the GLRLM feature category of the CT were reproducible by the radiomics features of CBCT. The other two feature categories of the CT, namely GLCM and NID, which were considered not reproducible across scanners even under idealized circumstances remained the same for the CBCT radiomics features. 

*ICC was calculated to measure the similarities of each feature category between the seven density materials of the CT and CBCT*

This analysis demonstrated reproducible feature category from different density image segments among the four scans. ICC of the images in the dissimilar mediums of four selected feature categories was compared in box plot wispier. In this comparison, the interquartile range of NID differed from the other three feature categories. The density materials of NID, namely Acrylic and Air, ROIs ICC= 0.418, P=0.209, ICC=0.607, P=0.09 demonstrating non-significant p- value . This is because of less uniformity in ROIs physical density (Fave et al., 2015). NID texture features are calculated from the spatial distribution of voxel intensities or CT numbers. Moreover, CBCT is an ancillary modality and considered for image guidance, its pixel units values are likely less accurate than CT pixel units and CBCT pixel mapping differs from CT images. In addtion, CBCT image formation method is different from CT modality. 

In a nutshell, 39% of the 49 selected computed features had strong and significant correlation with CT. While the remaining 61% features, had low and medium correlation. These findings further substantiated the probabilities of interrelation amongst the imaging modalities of discrete density material textures, thereby incrementing the reliability of our study. According to the ICC results, the four feature categories, namely intensity, GLCM, and GLRLM, showed significance/significant p value. This range of reproducible features can be helpful to provide unique information from anatomical tissues. However, the clinical studies showed different outcomes as compared to phantom study. Therefore, further studies correlating clinical with phantom studies would probably clarify the present limitations and possibly aid in better understanding of the applications of CBCT radiomics. In conclusion, as CBCT images are taken regularly, we had considered it for our study. According to findings of this phantom study, CBCT radiomics could probably be considered as an independent modality. 

## Author Contribution Statement

The authors confirm contribution to the paper as follows: study conception and design: Dharmenrdan palani 1^st^ Author; data collection: Dharmendran palani 1^st^ Author, Kesavan Govindaraj 3^rd^ Author ; analysis and interpretation of results: Dharmenran palani 1st Author, Senthilkumar Shanmugam 2^nd ^Author, Kesavan Govindaraj 3^rd^ Author; draft manuscript preparation: Dharmendran palani 1st Author, Senthilkumar Shanmugam 2^nd^ Author. All authors reviewed the results and approved the final version of the manuscript. 
